# Over-Expression of Rice CBS Domain Containing Protein, OsCBSX3, Confers Rice Resistance to *Magnaporthe oryzae* Inoculation

**DOI:** 10.3390/ijms160715903

**Published:** 2015-07-13

**Authors:** Shaoliang Mou, Lanping Shi, Wei Lin, Yanyan Liu, Lei Shen, Deyi Guan, Shuilin He

**Affiliations:** 1 College of Life Science, Fujian Agriculture and Forestry University, Fuzhou 350002, China; E-Mails: moushaoliang@163.com (S.M.); linweifafu@163.com (W.L.); 2 National Education Minster Key Laboratory of Plant Genetic Improvement and Comprehensive Utilization, Fujian Agriculture and Forestry University, Fuzhou 350002, China; E-Mails: lpsh2015@126.com (La.S.); liuyanyan910628@163.com (Y.L.); shorttubelycoris07@163.com (Le.S.); 000q010036@fafu.edu.cn (D.G.); 3 College of Crop Science, Fujian Agriculture and Forestry University, Fuzhou 350002, China

**Keywords:** rice, *Magnaporthe oryzae*, CBS domain containing proteins, immunity

## Abstract

Cystathionine β-synthase (CBS) domain containing proteins (CDCPs) constitute a big family in plants and some members in this family have been implicated in a variety of biological processes, but the precise functions and the underlying mechanism of the majority of this family in plant immunity remain to be elucidated. In the present study, a CBS domain containing protein gene, *OsCBSX3*, is functionally characterized in rice resistance against *Magnaporthe oryzae* (*M. oryzae*). By quantitative real-time PCR, transcripts of *OsCBSX3* are up-regulated significantly by inoculation of *M. oryzae* and the exogenously applied salicylic acid (SA) and methyl jasmonate (MeJA). *OsCBSX3* is exclusively localized to the plasma membrane by transient expression of OsCBSX3 fused to green fluorescent protein (GFP) through approach of *Agrobacterium* infiltration in *Nicotiana benthamiana* leaves. The plants of homozygous T_3_ transgenic rice lines of over-expressing *OsCBSX3* exhibit significant enhanced resistance to *M. oryzae* inoculation, manifested by decreased disease symptoms, and inhibition of pathogen growth detected in DNA. Consistently, the over-expression of *OsCBSX3* enhances the transcript levels of immunity associated marker genes including *PR1a*, *PR1b*, *PR5*, *AOS2*, *PAL*, *NH1*, and *OsWRKY13* in plants inoculated with *M. oryzae*. These results suggest that *OsCBSX3* acts as a positive regulator in resistance of rice to *M. oryzae* regulated by SA and JA-mediated signaling pathways synergistically.

## 1. Introduction

As sessile organisms, plants are continuously encountered by attacks of different potentially pathogentic microbes, and have developed defense systems to cope with these pathogens. Data from transcriptomics and proteomics studies show that a great many genes or proteins are involved in the response of plants to pathogen attack [1,2,3,4,5]. Although a zigzag concept model including pathogen-associated molecular pattern (PAMP)-triggered immunity (PTI) and effector-triggered immunity (ETI), which extensively share signaling machinery, have been established [6,7,8,9,10,11], the majority of the components and their precise role in plant inducible immunity remain uninvestigated. Functional characterization of these components may lead to the discovery of novel or alternate pathways.

Cystathionine β-synthase (CBS) domain, firstly identified in the genome of an archaebacterium *Methanococcus jannaschii* [12], has also been reported in yeast and animal systems that play roles in proteins of diverse functions including cytoplasmic targeting, subcellular localization of chloride channels (ClC), protein–protein interaction, protein regulation, sensors of cellular energy status, and intracellular ionic strength [12]. CBS domain containing proteins (CDCPs) have been found in eubacteria and eukaryotes [13], which containing CBS domains generally in tandem repeats, form a gene family with 34 and 59 members in *Arabidopsis* and rice, respectively [14]. AKINbc, a CDCP of *Arabidopsis* containing four CBS domains, interacted with AKINa1 and a2 kinases and its fourth CBS domain was found to be essential but not sufficient for interaction with kinases [15]. CBS domain containing proteins (CBSX1 CBSX2 and CBSX3) were found to be ubiquitous redox regulators that regulate thioredoxins in the ferredoxin-Trx system and NADP (nicotinamide adenine dinucleotide phosphate)-Trx system to modulate development and maintain homeostasis under conditions that are threatening to the cell [16]. Hybrid four-CBS-domain KINβγ subunit functions as the canonical gamma subunit of the plant energy sensor SnRK1 (SNF1-related kinase 1) [17]. OsCBSX4, a CBS domain containing protein in rice, was found that its over-expression in transgenic tobacco plants improved tolerance to salt, heavy metal, and oxidative stress [18]. OsBi1 encoding a protein containing CBS-like domain was found to be induced by herbivore feeding [19]. By transcriptomic and proteomic studies, proteins containing CBS domains exhibited differential expressional profiles in plants challenged with virus [20], fungi [21], salinity stress [22,23], and oxalic acid treatment [24]. All these data indicate that the members in this family in different plant species may play important roles in plant response to stresses. However, so far only a small number of CBS domain containing proteins have been functionally identified, the majority members in this family remain uninvestigated, especially in their role in plant immunity.

Rice (*Oryza sativa*) is one of the most important crops worldwide. Rice blast, caused by *Magnaporthe oryzae*, is the most destructive disease in rice production in China and other rice-growing regions [25]. The fungus is a filamentous ascomycete with a broad host range and leads to critically decrease in both rice yield and quality [26], and it destroys enough rice to feed more than 60 million people annually [27]. For decades, efforts to solve the problem caused by this disease have been focused on molecular mechanism studies of blast resistance, which may benefit the development of blast resistant rice varieties. However, the knowledge in this field is still limited. In the present study, we describe that a rice CDCP gene *OsCBSX3*, which is transcriptionally up-regulated by *M. oryzae*, acts as positive regulator in the response of rice to *M. oryzae* in a salicylic acid and jasmonate dependent manner.

## 2. Results

### 2.1. The Sequence Analysis of OsCBSX3 (Cystathionine β-Synthase (CBS) Domain Containing Protein 3)

**Figure 1:**
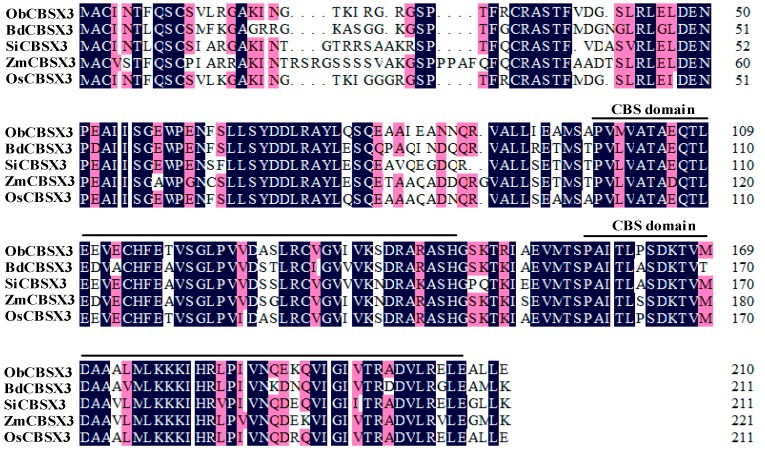
Multiple sequence alignment of the amino acid sequence of OsCBSX3 (cystathionine β-synthase (CBS) domain containing protein 3) and its homolog protein. The CBS domain is underlined predicted by SMART server (http://smart.embl-heidelberg.de/). OsCBSX3 homolog proteins are from *Oryza brachyantha* (*ObCBSX3*), *Setaria italica* (*SiCBSX3*), *Brachypodium distachyon* (*BdCBSX3*) and *Zea mays* (*ZmCBSX3*). Sequeneces identical or similar are highlighted in black and red, respectively.

A TDF (transcript derived fragment), which harbored two CBS domains, was found previously in our lab by cDNA-AFLP (restriction fragment length polymorphism) analysis of rice against the inoculation of *M. oryzae*. For cDNA-AFLP analysis, 16 pairs of primers were used for selective PCR amplification and total 12,000 TDFs were acquired. Among them, 240 differential TDFs were isolated and sequenced. In the present study, the full length cDNA of this TDF was identified by genome sequence search, and was further cloned by PCR amplification from cDNA library of Nipponbare with specific primers according to the full length cDNA, which was further cloned to pMD18-T vector and sequenced. The result showed that the full length of *OsCBSX3* cDNA harboring an open reading frame 639 bps in length encoded a protein with 213 amino acids, which accordingly contained two CBS domains by SMART server (http://smart.embl-heidelberg.de/). Alignment analysis of the deduced amino acid sequence of the full length cDNA revealed that it shared 92.9%, 79.3%, 79.3%, and 75.2% identity with *Oryza brachyantha* (LOC102720769), *Setaria italica* (LOC101778698), *Brachypodium distachyon* (LOC100834804) and *Zea mays* (LOC100282160) (Figure 1), respectively.

### 2.2. Transcript Levels of OsCBSX3 Enhanced in Response to M. oryzae Inoculation and Exogenous Application of Salicylic Acid (SA) and Methyl Jasmonate (MeJA)

**Figure 2:**
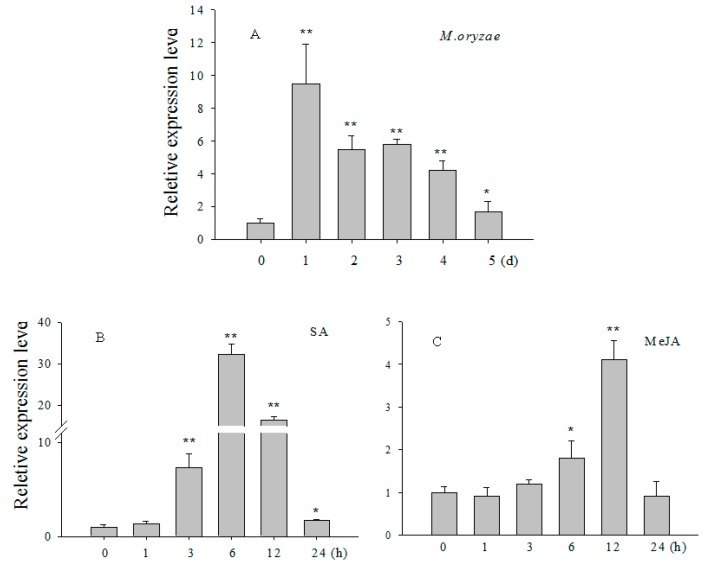

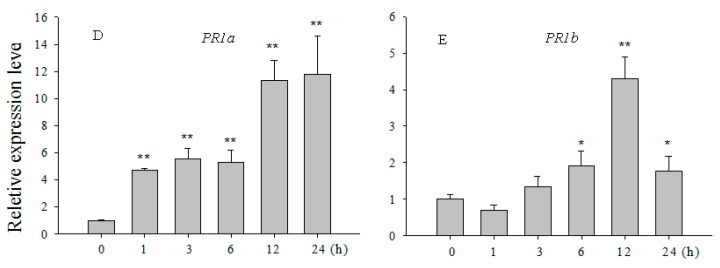
Quantitative PCR analysis of relative *OsCBSX3* transcript levels in rice plants exposed to pathogens and exogenous hormones.(**A**) *OsCBSX3* transcripts tested at different time points in the rice leaves after inoculation with *M. oryzae* strain guy11; (**B**–**E**) *OsCBSX3* transcripts examined in rice leaves at various time periods after treatment with salicylic acid (SA) (100 μM) or methyl jasmonate (MeJA) (100 μM). *OsPR1a* or *OsPR1b* genes were used as positive controls, respectively. (**A**–**E**) Transcript levels of *OsCBSX3* or marker genes in pathogen- or hormone-treated rice plants were normalized to those in mock-treated control plants, which were set to a relative expression level of “1”. Error bars indicate the standard error; the experiments were repeated three times along with at least three independent repetitions of the biological experiments. Asterisks indicate significant differences (Student–Newman–Keuls test, * *p* *<* 0.05 or ** *p* *<* 0.01).

To test if *OsCBSX3* was involved in pathogen defense, *OsCBSX3* transcript levels were measured in rice leaves of Nipponbare after inoculation with strain guy11 of *M. oryzae*. Transcript levels were measured by real-time PCR at different dpi (days post inoculation). The result showed that transcript levels of *OsCBSX3* enhanced significantly during 1 to 5 dpi of *M. oryzae*, with a rapid increase of approximate 9.5-fold at 1 dpi compared to that in mock-treated control plants. The effects of exogenous application of SA and methyl jasmonate (MeJA) in rice plants were detected by real time PCR with SA-dependent marker gene *PR1a* and JA-dependent basic PR1 (*PR1b*) gene [28,29]. The results showed that, in response to exogenously applied 100 µM SA, transcripts of *OsCBSX3* enhanced significantly during 3–24 hpt (hours post treatment), with maximal levels of approximately 32.2-fold at 6 hpt compared to that in mock-treated control plants. After treatment with 100 µM MeJA, *OsCBSX3* transcripts enhanced approximately 1.8- and 4.1-fold at 6- and 12-hpi, compared to that in mock-treated control plants (Figure 2).

### 2.3. OsCBSX3 Is Localized in the Plasma Membrane

**Figure 3:**
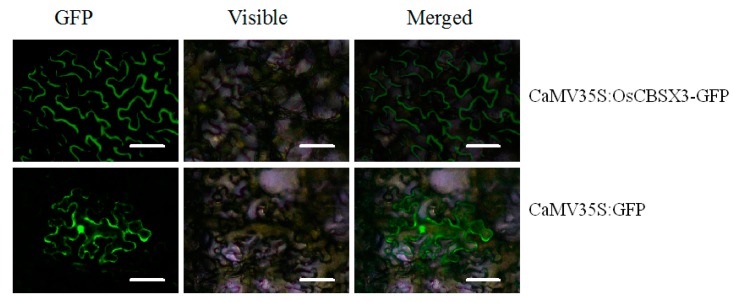
Subcellular localization of OsCBSX3 protein in *N. benthamiana* leaves. OsCBSX3-GFP (green fluorescent protein) exclusively localized in the plasma membrane of cell in *N. benthamiana* leaves. GFP alone localized throughout the whole cells. Cells were detected for GFP fluorescence by fluorescence microscopy 48 h after agroinfiltration. Scale bars =10 µm.

To determine the subcellular localization of OsCBSX3, *OsCBSX3* was fused to the green fluorescent protein (GFP) gene under control of 35S promoter, the resulting construct was transformed into *Agrobacterium* strain GV3101 and infiltrated into *N. benthamiana* leaves. The GFP signal was detected with fluorescence microscope (Olympus DP72), the result showed that the GFP signal was exclusively found in the plasma membrane (Figure 3).

### 2.4. Over-Expression of OsCBSX3 in Transgenic Rice Plants Conferred Enhanced Resistance to M. oryzae Inoculation

**Figure 4:**
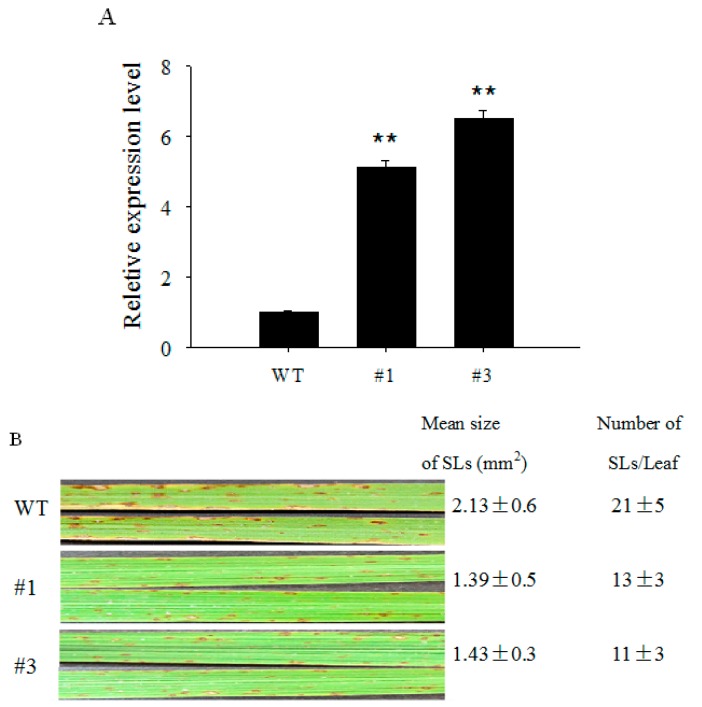

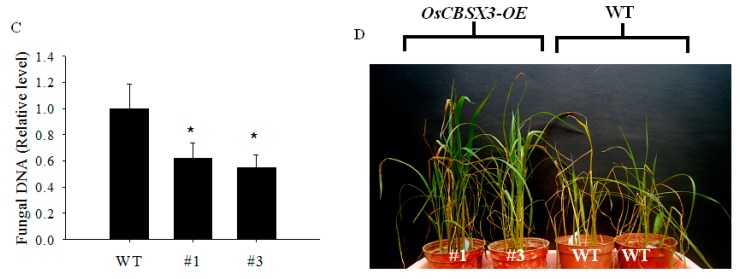
Over-expression of *OsCBSX3* enhanced rice resistance to blast fungus. (**A**) Quantitative PCR analysis of *OsCBSX3* expression in wildtpye (WT) and over-expression plants (#1 and #3). Data are means ± SE with three independent experiments. Asterisks indicate significant differences (Student–Newman–Keuls test, * *p* *<* 0.05 or ** *p* *<* 0.01); (**B**) Lesion in leaves at 6 dpi (days post inoculation). Numbers of expanding lesions (Els) with an area greater than 0.5 mm^2^ per leaf and their mean areas were determined using ten leaves for WT and *OsCBSX3*-*OE* plants. Values given are means ± SE; (**C**) The amounts of *M. oryzae* DNA in the WT and *OsCBSX3*-*OE* rice leaves. The leaves were harvested at 6 dpi. Values were means ± SE of three independent experiments; (**D**) Symptom of rice blast in *OsCBSX3*-*OE* and WT rice plants at 30 dpi of spores of *M. oryzae* stain guy11 in greenhouse.

Since *OsCBSX3* transcripts are transcriptionally upregulated by *M. oryzae* inoculation and by exogenous applied SA and MeJA. It appeared that *OsCBSX3* plays a role in rice immunity against *M. oryzae* attack. To confirm this possibility, we generated transgenic rice T_3_ homozygous lines constitutively expressing *OsCBSX3* driven by the maize ubiquitin promoter. A total of sixteen T_3_ lines were acquired, and no phenotypic difference was observed between *OsCBSX3-OE* (over-expression) T_3_ lines and wildtype (Nipponbare) rice plants. Among the sixteen lines, two higher expression lines (lines #1 and #3) were chosen for further assay (Figure 4A). *M. oryzae* strain guy11 was used to evaluate the resistance of the *OsCBSX3-OE* lines and wildtype plants to rice blast disease. All of the rice plants at four-leaf stage were inoculated with guy11, and grown in a greenhouse. The rice blast symptoms were observed at 5 dpi, the two *OsCBSX3-OE* lines showed fewer and smaller expanding lesions compared to the wild type plants (Figure 4B). The growth of *M. oryzae* was detected by measuring the total DNA of *M. oryzae* in the inoculated rice leaves, and the results showed that growth of *M. oryzae* was significantly inhibited in *OsCBSX3-OE* lines compared to that in the wild type plants (Figure 4C) Wild type rice plants began to wilt at 20 dpi, some plants were dead at 35 dpi. In contrast, all of *OsCBSX3-OE* rice lines survived with decreased disease symptoms compared to those in wild-type plants (Figure 4D).

### 2.5. Over-Expression of OsCBSX3 Up-Regulated the Transcript Levels of Defense Marker Genes

**Figure 5:**
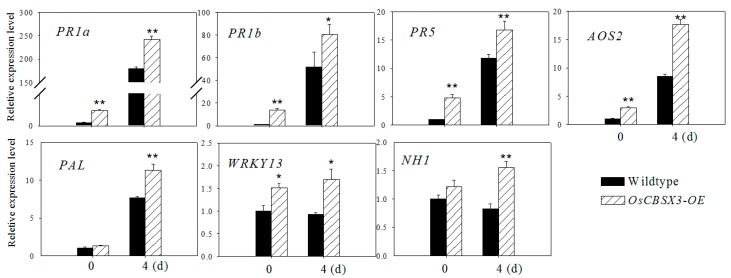
Quantitative PCR analysis of relative expression levels of pathogen-related genes in leaves of wildtype plants and *OsCBSX3* over-expression lines at 0 and 4 dpi of *M. oryzae* strain guy11. Bars represent mean ± SE of three biological replicates. Asterisks indicate significant differences between the *OsCBSX3-OE* and wildtype plants in the same time point (Student–Newman–Keuls test, * *p* *<* 0.05 or ** *p* *<* 0.01).

To further confirm the role of *OsCBSX3* in disease resistance and to assay its possible mode of action, the transcriptional response of a set of pathogen-induced genes in *OsCBSX3* over-expression rice plants were investigated by real-time PCR. These immunity marker genes including Phenylalanine ammonia lyase (*PAL*; X87946) involved in SA synthesis, Acidic pathogenesis-related (PR) protein 1 (*PR1a*; AJ278436), *PR5* (thaumatin-like protein; X68197), and *NH1* (*Arabidopsis* *NPR1* homolog 1; AY9123983) are response to SA [30,31,32,33]. JA associated Allene oxide synthase 2 (*AOS2*; AY062258) [34] and *PR1b* (basic PR protein 1; U89895) [30,32] as well as *OsWRKY13*, a WRKY transcriptional factor that act as positive regulators in rice immunity by previous study [30]. Our data showed that the transcript levels of *PR1a*, *PR1b*, *PR5*, *AOS2*, and *PAL* were increased in *OsCBSX3* over-expression plants in response to *M. oryzae* infection, the transcripts of *PR1a*, *PR1b*, *PR5*, *AOS2* accumulated to significantly higher levels with and without *M. oryzae* inoculation in *OsCBSX3* over-expression plants compared to those in wild-type plants, *PAL* and *NH1* were only enhanced by over-expression of *OsCBSX3* with *M. oryzae* inoculation (Figure 5).

## 3. Discussion

Although CDCPs constitute a big family in plants and some members in this family have been implicated in a variety of biological processes, the precise functions and the underlying mechanism of the majority of this family in plant immunity remain to be investigated. In the present study, we provide evidence that *OsCBSX3,* a member of CDCPs in rice, plays a positive role in the response of rice to *M. oryzae* attack. Moreover, *OsCBSX4* over-expressing transgenic tobacco plants exhibit higher abiotic stress tolerance [18]. Microarray expression data showed that some members of this family were transcriptionally altered in response to various stresses such as salinity, drought, cold, high temperature, UV, wounding and genotoxic stress [14]. So CDCPs might play an important role in biotic or abiotic stress tolerance.

Two CBS domains forming a pair were found to be present in the deduced amino acid sequence of *OsCBSX3*, and a high sequence similarity to other CBS domain containing proteins from rice or other plants was also found, suggesting that it is a member of the CDCP family in rice. However, unlike AtCBSX3 in *Arabidopsis* that was predicted to localize to the mitochondria, AtCBSX4 to the cytosol [16], OsCBSX3 is found to target to the plasma membrane. Consistently, the OsCBSX4 in rice was also found to be localized to the plasma membrane [18]. The transcriptionally up-regulation of *OsCBSX3* by the *M. oryzae* inoculation implies its functional association to rice’s response to *M. oryzae* attack, since genes involved in plant immunity generally possess pathogen inducible expressional features and a close relationship has been frequently found in the expression profiling and the role in immunity of a great number of immunity associated genes [35,36]. The possible role of *OsCBSX3* in rice immunity against *M. oryzae* is further confirmed by gain of function analysis using *OsCBSX3* over-expressing rice plants of homozygous T_3_ transgenic lines, the result demonstrates that the susceptibility of rice plants to *M. oryzae* attack is significantly decreased by over-expression of *OsCBSX3*, manifested by decreased disease symptoms, inhibition of pathogen growth. Consistently, *PR1a*, *PR1b*, *PR5*, *AOS2*, *PAL*, and *NH1*, which have been found to act as positive regulators in rice immunity and generally used a marker genes in rice immunity [30,32], are found to be enhanced in *M. oryzae* challenged *OsCBSX3* over-expressing transgenic rice plants. Os*WRKY13*, over-expression of which enhanced rice resistance to bacterial blight and fungal blast [35], is also found to be enhanced by the over-expression of *OsCBSX3.* All of these data suggest that *OsCBSX3* acts as positive regulator in response of rice to *M. oryzae* attack.

Plant defense against pathogens form a comprehensive network of interacting signal transduction pathways, the signaling molecules salicylic acid (SA) and jasmonic acid (JA) play important roles in this network. Both synergistic and antagonistic interactions have been observed between SA- and JA-dependent defenses [37,38,39]. Our data showed that the expression of *OsCBSX3* is transcriptionally induced by exogenous application of SA and MeJA. Consistently, SA-dependent *PR1a* and JA responsive *PR1b* are enhanced by the over-expression of *OsCBSX3* in transgenic rice plants, suggesting that the defense reaction mediated by *OsCBSX3* is SA and JA synergistically dependent. This speculation is consistent with the data that *OsWRKY13* is triggered by the over-expression of *OsCBSX3*, and with previous study, *OsWRKY13* was synergistically regulated by SA- and JA-dependent signaling pathways [30].

Interestingly, the immunity associated genes such as *NH1* and *PAL* are only transcriptionally enhanced by over-expression of *OsCBSX3* under the challenge of *M. oryzae* inoculation, but not in the *M. oryzae* un-challenged transgenic plants. It suggests that the transcriptional upregulation of these genes need other component that is activated by *M. oryzae* inoculation. Additionally, although the transcript level of *OsCBSX3* in *OsCBSX3*-*OE* lines is lower than that in the wild type plants during *M. oryzae* infection, the *OsCBSX3*-*OE* lines exhibited significantly enhanced resistance to *M. oryzae* inoculation, suggesting that a high transcript level of *OsCBSX3* prior to *M. oryzae* infection is important for rice resistance to *M. oryzae*.

Collectively, our data in the present study show that *OsCBSX3* are upregulated transcriptionally by *M. oryzae* attack and acts as a positive regulator in rice resistance to *M.* *oryzae*.

## 4. Experimental Section

### 4.1. Plant Growth and Treatments

Rice (*Oryza sativa* L. japonica) Nipponbare and *Nicotiana benthamiana* seedlings were grown in a greenhouse at Fujian Agriculture and Forestry University (Fuzhou, China) under 25/27 °C (night/day). For inoculation of rice plants with the pathogen, three-week-old seedlings leaves were sprayed with a suspension of conidia (1 × 10^5^ mL^−1^) of *Magnaporthe oryzae* strain guy11. For exogenous phytohormone treatment, SA (100 µM in 10% ethanol), or 100 µM MeJA (in 10% ethanol) was sprayed on the leaves. All experiments were performed independently three times, and the data from these experiments were subjected to statistical analysis using DPS software (Reifeng information technology Corporation, Hangzhou, China).

### 4.2. Cloning, Plasmid Construction and Rice Transformation

The *OsCBSX3* gene (LOC_Os02g57280) cDNA was PCR amplified using cDNA library from rice (*Oryza sativa* L. japonica) Nipponbare leaves inoculated with guy11 as template with *CBSX3F* (5′-ATGGCCTGCATCAACACA-3′) and *CBSX3R* (5′-CTAAACCTCCAGCAGGGC-3′) primers, the amplified product was further cloned into the pMD18-T vector and sequenced. *OsCBSX3* cDNA was amplified by PCR with *Kpn* I–*Spel* I linker primers using pMD18T-OsCBSX3 as the template, which was further cloned into the *Kpn* I and *Spel* I sites of the modified binary expression vector pCAMBIA1390 under the control of the maize ubiquitin promoter. The transgenic rice plants were obtained by *Agrobacterium*-mediated transformation [40] and confirmed by PCR with specific primer. The T_0_ transgenic rice plants were grown in the greenhouse and the seeds were harvested. The transgenic T_1_ seeds were selected with 50 mg/L hygromycin and grown in greenhouse for seeds of individual T_2_ lines, which was selected with hygromycin and grown to harvest for seeds of individual T_3_ lines.

### 4.3. RNA Isolation and Quantitative Real-Time PCR (qRT-PCR)

Total RNA from rice leaves was isolated using the TRIzol Reagent (Invitrogen, Carlsbad, CA, USA). Moloney Murine Leukemia Virus (M-MLV) Reverse Transcriptase (Invitrogen) was used for cDNA synthesis according to the manufacturer’s protocol. Real-time PCR using Mastercycler ep realplex (Eppendorf, Hamburg, Germany) was performed with SYBR^®^ Premix Ex Taq™ II (TaKaRa, Dalian, China). Each reaction (25 μL) consisted of 12.5 μL SYBR Premix Ex Taq, 0.5 μL PCR forward/reverse gene specific primers (10 μM) 2.5 μL diluted cDNA and 9.5 μL water. qRT-PCR cycling conditions were as follows: 1 cycle of 30 s at 95 °C; 40 cycles of 5 s at 95 °C, 34 s at 60 °C; 1 cycle of 15 s at 95 °C, 1 min at 60 °C, 15 s at 95 °C, 15 s at 60 °C. For each gene, three experimental replicates were obtained using different cDNAs synthesized from at least three biological replicates. To normalization the variance in the RNA quality and cDNA input, rice Actin gene (X15865) was used as the internal control in each case. The relative expression levels were determined as described by Livak and Schmittgen [41].

**Table 1:** Primers information for real-time PCR.

Gene Name	Forward Primer Sequence (5′–3′)	Reverse Primer Sequence (5′–3′)
*CBSX3*	GAGGAGGTTGAGTGCCACTTTG	GCCGCATCCATTACTGTTTTGTC
*PR1a*	CGTCTTCATCACCTGCAACTACTC	CATGCATAAACACGTAGCATAGCA
*PR1b*	GGCAACTTCGTCGGACAGA	CCGTGGACCTGTTTACATTTTCA
*PR5*	CAACAGCAACTACCAAGTCGTCTT	CAAGGTGTCGTTTTATTCATCAACTTT
*NH1*	CACGCCTAAGCCTCGGATTA	TCAGTGAGCAGCATCCTGACTAG
*WRKY13*	TCAGTGGAGAAGCGGGTGGTG	GGGTGGTTGTGCTCGAAGGAG
*AOS2*	CAATACGTGTACTGGTCGAATGG	AAGGTGTCGTACCGGAGGAA
*PAL*	AGCACATCTTGGAGGGAAGCT	GCGCGGATAACCTCAATTTG
*Actin*	TGTATGCCAGTGGTCGTACCA	CCAGCAAGGTCGAGACGAA

### 4.4. Subcellular Locatization

*OsCBSX3* cDNA without its stop codon was amplified and fused in frame to the N-terminus of the green fluorescent protein (GFP) gene to generate the CaMV35S:OsCBSX3-GFP construct by Gateway-mediated recombination into the vector pMDC83 [42]. The CaMV35S:GFP construct was used as the control. Both constructs were further transformed into *Agrobacterium tumefaciens* strain GV3101. *Nicotiana benthamian* leaves were infiltrated with *Agrobacterium* cultures. One day after agro-infiltration, Green fluorescent protein fluorescence was visualized using a fluorescence microscope (Olympus DP72, Olympus Corporation, Tokyo, Japan) with an excitation wavelength of 488 nm and a 505–530 nm bandpass emission filter.

### 4.5. Quantification of M. oryzae DNA in Rice Leaves

Infected rice leaves were detached at five days post-inoculation of *Magnaporthe oryzae*, rice and fungal DNA were extracted and quantified by real-time PCR according to the method of Qi and Yang [43]. A specific pair of DNA primers (5′-GGGATGATGGTGGTGGAGGAC-3′; 5′-GCCAGGTGCTTAGGACGAAAC-3′) were designed based on the 3′ non-coding region of a *MPG1* gene in *M. oryzae*. The data were normalized to the amount of DNA of a rice actin gene (AK060893), which was quantified using the forward primer (5′-GAGTATGATGAGTCGGGTCCAG-3′) and reverse primer (5′-ACACCAACAATCCCAAACAGAG-3′).

## 5. Conclusions

A CBS domain containing protein gene, *OsCBSX3*, was identified and its involvement in rice resistance against *M. oryzae* was characterized. *OsCBSX3* gene expression was up-regulated significantly by inoculation of *M. oryzae* and the exogenously applied salicylic acid (SA) and methyl jasmonate (MeJA). OsCBSX3 was exclusively localized to the plasma membrane. The transgenic plants of *OsCBSX3* over-expression exhibited significant enhanced resistance to *M. oryzae* inoculation. Consistently, the over-expression of *OsCBSX3* enhanced the transcript levels of immunity associated marker genes including *PR1a*, *PR1b*, *PR5*, *AOS2*, *PAL*, *NH1*, and *OsWRKY13*. These results suggested that *OsCBSX3* acted as a positive regulator in rice resistance to *M. oryzae*. Our work will help us better understand the molecular mechanisms in rice resistance to *M. oryzae*.
